# Effects of autophagy on macrophage adhesion and migration in diabetic nephropathy

**DOI:** 10.1080/0886022X.2019.1632209

**Published:** 2019-07-29

**Authors:** Yuteng Jiang, Yu Zhao, Xiaodong Zhu, Yuqiu Liu, Beibei Wu, Yinfeng Guo, Bicheng Liu, Xiaoliang Zhang

**Affiliations:** Institute of Nephrology, Zhong Da Hospital, Southeast University, School of Medicine, Nanjing, Jiangsu, China

**Keywords:** Macrophages, high glucose, autophagy, adhesion, migration

## Abstract

**Objective:** Macrophage infiltration in kidney is a major pathological feature of diabetic nephropathy (DN), which has been demonstrated associate with macrophages autophagy homeostasis. However, the relationships between autophagy and the infiltration response related of macrophages adhesion and migration are unknown. This study aims to investigate the impact of macrophages adhesion and migration by modulating autophagy.

**Methods:** In vivo, rats were randomly distributed into control (NC) and DN groups. The pathological changes in renal tissue were assessed, and expression of CD68, LC3, P62 were analyzed. In vitro, RAW264.7 cells were divided into NC and high glucose (HG) groups. The capacity of macrophages adhesion migration and the expression of autophagy markers were observed with and without autophagy modulators (rapamycin, 3-methyladenine, chloroquine, and bafilomycin A1 for RAPA, 3-MA, CQ, BAFA). The macrophages autophagosome and the process of degradation and fusion of autophagosome-lysosome were observed by electron microscopy.

**Results:** In vivo, renal injury is aggravated in diabetic rat compared with NC group. The autophagy level is inhibited in renal tissues of DN group with the increasing expression of CD68 and P62, while expression level of LC3 decreased (*p* < .05). In vitro, HG and 3-MA reduce the numbers of autophagosome of macrophages to inhibit autophagy level with decrease expression of LC3 and Beclin-1, but increase expression of P62, which promote the adhesion and migration capacity of macrophages (*p* < .05). Moreover, CQ and BAFA suppress autophagy level by inhibiting the process of autophagosome-lysosome degradation and fusion of macrophages, as well as the expression of LC3 and Beclin-1. We notice an increase expression of P62 by CQ and BAFA stimulation (*p* < .05). CQ and BAFA further facilitate the adhesion and migration capacity of macrophages. However, RAPA increases the numbers of macrophages autophagosome that inhibited by HG, resulting in a recovery of autophagy level with increase expression of LC3 and Beclin-1, whereas a reduction expression of P62, which lead to inhibition of adhesion and migration of macrophages induced by HG (*p* < .05)

**Conclusions:** High glucose efficiently reduced the level of macrophage autophagy, following macrophages adhesion and migration enhanced when autophagy is suppressed. Activation of autophagosome improve the level of autophagy, but leading to a reduction of the macrophages adhesion and migration. While, inhibiting the process of degradation and fusion of autophagosome-lysosome suppress the level of autophagy and promote the macrophages adhesion and migration. These results indicate that high glucose may play an important role in macrophages adhesion and migration through modulating autophagy activities in diabetic nephropathy.

## Introduction

Diabetic nephropathy (DN) is a chronic inflammatory disease that is involved in leukocyte infiltration, cell proliferation, and extracellular matrix accumulation [1]. Autophagy is a physiological degradation and reuse process that is mediated by cellular lysosomes. This process degrades cytoplasmic components, including misfolded proteins and dysfunctional organelles, to deal with different stress conditions and maintain the normal function of the cell [2–5]. Macrophages, a type of important inflammatory and immune cell, participate in inflammation, damage, fibrosis, repair and other different disease stages to maintain homeostasis [6,7]. Macrophages can migrate to areas of inflammation, where they gather, secrete cytokines and play a role in phagocytosis. Therefore, it is important to reduce macrophage adhesion and migration to prevent DN. This study aims to investigate the effects of down-regulating and recovering autophagy levels on macrophage adhesion and migration in cell experiments under high-glucose conditions.

## Materials and methods

Healthy male rats, 6 weeks old, body mass of 180 ∼ 220 g, purchased from Shanghai SLAC, Ltd (Shanghai, China); Streptozotocin (STZ, Sigma, St. Louis, MO, USA); Glucose meter (Roche, Mannheim, Germany); CD68 (Santa Cruz Biotechnology, Santa Cruz, CA, USA); RAW264.7 cells (Shanghai Bogu Biotechnology Co, Shanghai, China); RPMI-1640 medium (Gibco, Waltham, MA, USA); fetal bovine serum (ScienCell, Carlsbad, CA, USA); Dextrose, mannitol, and Rapamycin (RAPA; Sigma, USA); 3-methyladenine (3-MA) (Sigma, USA); Bafilomycin A1 (BAFA; Abcam, Cambridge, UK); Chloroquine (CQ; Sigma, USA); CD68 (Abcam); LC3 (Sigma, USA); Beclin 1 (Abcam); P62 (Cell Signaling Technology, Danvers, MA, USA); Transwell Chambers (Sigma, USA); and Fibronectin (FN; Cloud Clone, Katy, TX, USA).

### Establish the model of diabetic rats

All animal care and experimental protocols were in compliance with the Animal Management Rules of the Ministry of Health of the People’s Republic of China. Six-week-old healthy male Sprague–Dawley (SD) rats weighing 200 ∼ 220 g were obtained from Shanghai SLAC Laboratory Animal Company, Ltd. After one week of acclimation, the rats were randomly divided into two groups: NC (normal control group, *n* = 6) and DN (DN rats, *n* = 6). DN was induced with a single intraperitoneal injection of STZ (Sigma) dissolved in 0.1 M citrate buffer (pH 4.5) at 58 mg/kg, while the control rats received only the 0.1 M citrate buffer solution. Three days later, the diabetic state was confirmed by measuring the tail blood glucose (BG) level. Rats with a BG level over 16.7 mmol/L were considered diabetic rats. BG was monitored with a BG monitoring system (Bayer, Leverkusen, Germany) using one drop of tail blood. Twenty-four hour urine samples were collected in metabolic cages. Blood samples were taken to measure biochemical parameters, and kidneys were collected for histological examination and molecular assays.

### Renal pathological examination

The renal tissue was embedded in conventional paraffin and was cut into 2 µm thick slices with HE, PAS, and Masson staining. Under the light microscope (×400), the glomerular size, basement membrane and mesenchymal hyperplasia were observed, and 20 intact glomeruli were randomly selected from each slice.

### Culturing and grouping

The mouse macrophage cell line RAW264.7 was purchased from Shanghai Bogoo Biotechnology Company and was routinely cultured in RPMI 1640 media (containing 11.1 mM glucose) supplemented with 10% fetal bovine serum (Sciencell, Santiago, MN, USA) and incubated at 37 °C in 5% CO_2_. When the cells reached 70%∼80% confluence, they were inoculated on a six-well plate. RAW264.7 cells were classified into 11 groups corresponding to the in vivo control group (NC group, 11 mmol/L glucose), high-glucose group (HG group, 30 mmol/L glucose), 3-MA group (11 mmol/L glucose + 1 mmol/L 3-MA), HG + RAPA group (30 mmol/L glucose + 10 nmol/L RAPA), HG + 3-MA group (30 mmol/L glucose + 1 mmol/L 3-MA), HG + CQ group (30 mmol/L glucose + 50 µmol/L CQ), HG + BAFA group (30 mmol/L glucose + 0.5 µmol/L BAFA), and mannitol group (11 mmol/L mannitol + 19 mmol/L mannitol). All the above groups were cultured for 24 h, and the experiment was repeated at least three times.

### Migration assays

Transwell migration assays were performed (Costar polycarbonate filters, 5 µm pore size). Macrophages (0.5 × 10^5^ cells/well) were added to the upper chamber without serum, and complete medium (10% serum) was added to the lower chamber. Cells migrating to the lower chamber after 8 h of incubation were washed, fixed in formaldehyde, stained with crystal violet, and then counted with Image Pro Plus 6.0.

### Adhesion assays

Fibronectin (Sigma) was used to coat 96-well plates overnight at 4 °C. Macrophages (0.1 × 10^5^ cells/plate) were added to the fibronectin-coated plates and incubated for 4 h at 37 °C. Adherent cells were then washed, fixed in formaldehyde, stained with Crystal violet, and then counted with Image Pro Plus 6.0.

### Western blot analysis

Cells were cultured for 24–48 h prior to lysis in buffer containing 50 mmol/l Tris HCl (pH 8.0), 150 mmol/l NaCl, 1% (v/v) Triton X-100 and a protease inhibitor cocktail (1:100 dilution; Sigma, Shanghai, China). The protein concentration was determined using Bradford reagent. The proteins were then separated by electrophoresis using 8.5% polyacrylamide gels and transferred onto nitrocellulose membranes. The membranes were subsequently exposed to Beclin-1 (1:1000), p62 (1:1000), CD68^+^ (1:1000), and LC3 (1:1000) antibodies and incubated at 4 °C overnight, prior to exposure to secondary antibody (Goat Anti-Rabbit IgG H&L; HRP; 1:1000) for 1 h at room temperature. β-actin (1:5000; Sigma) was used as a loading control. Membranes were developed using an enhanced chemiluminescence detection system (Denville Scientific, Holliston, MA, USA).

### Electron microscopy

The cells were fixed in ice-cold 2.5% glutaraldehyde in 0.1 M PBS and stored at 4 °C prior to further processing. The cells were postfixed in 1% osmium tetroxide in ice-cold 2.5% glutaraldehyde in 0.1 m PBS and then dehydrated through an alcohol series prior to embedding in Epon™ 812 (Electron Microscopy Sciences, Hatfield, PA, USA). The cells were next sectioned using an ultramicrotome (Leica, Wetzlar, Germany). Finally, the sections (500 nm) were stained with uranyl acetate and lead citrate and examined by transmission electron microscopy (Philips, Eindhoven, The Netherlands).

### Statistical analysis

All the experiments were repeated at least three times. The data are expressed as the mean and the standard deviation (SD) and were analyzed with SPSS 19.0. One-way ANOVA was used to evaluate multiple groups; the SNK test was adopted for two comparisons; and the correlation analysis between the two variables was analyzed by Pearson correlation. A difference was considered significant if the *P* value was <.05.

## Results

### Renal injury was aggravated in the diabetic rat model

**Figure 1.**
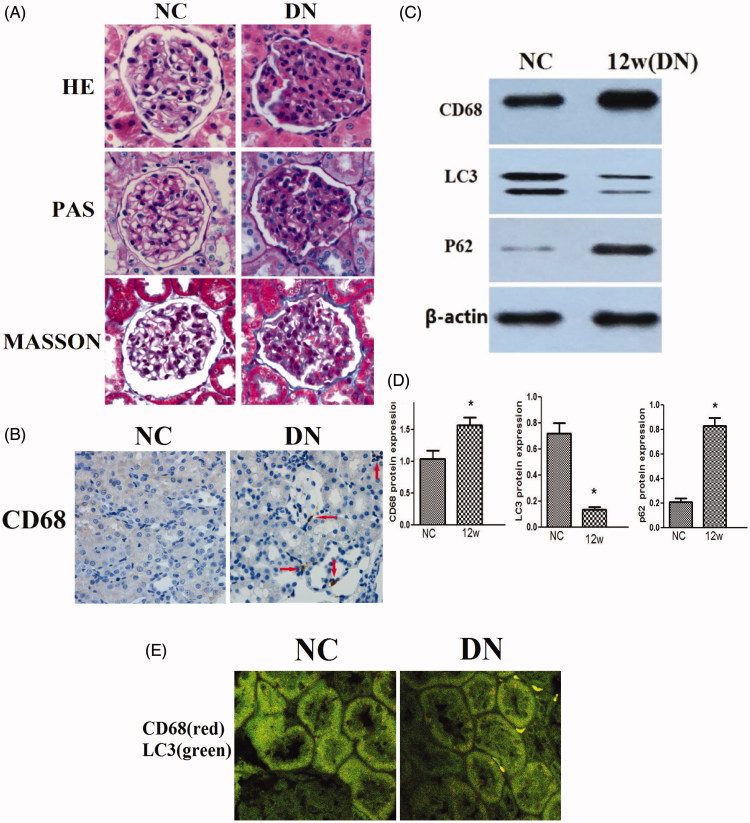
The pathological changes of kidney tissues and the expression of autophagy markers in rats in 12 weeks. (A) The pathological changes of kidney tissues of rats in 12 weeks (×400) (*n* = 6). (B) Renal macrophage CD68^+^ immunohistochemistry staining (×400). (C and D) Expression of CD68, LC3, and P62 protein (Western blotting), *p* < .05 versus NC (*n* = 3). (E) Immunofluorescence co-staining of macrophage marker CD68^+^ and LC3 in renal tissue.

Compared with those of the rats in the NC group, the volume of the glomeruli was increased, the basement membrane was thickened, and the mesangial matrix was increased in the kidney tissues of rats in the DN group (Figure 1(A)). Macrophage infiltration was increased in renal tissue of DN group showed by CD68, the macrophage maker (Figure 1(B)). Compared with NC group, the expression of macrophage marker CD68^+^ was increased in kidney tissue in DN group at 12 weeks, the expression of P62 was increased and the expression of LC3 was reduced (*P* < .05; Figure 1(C–E)).

### High glucose down-regulated autophagy level and promoted adhesion and migration of macrophages

**Figure 2.**
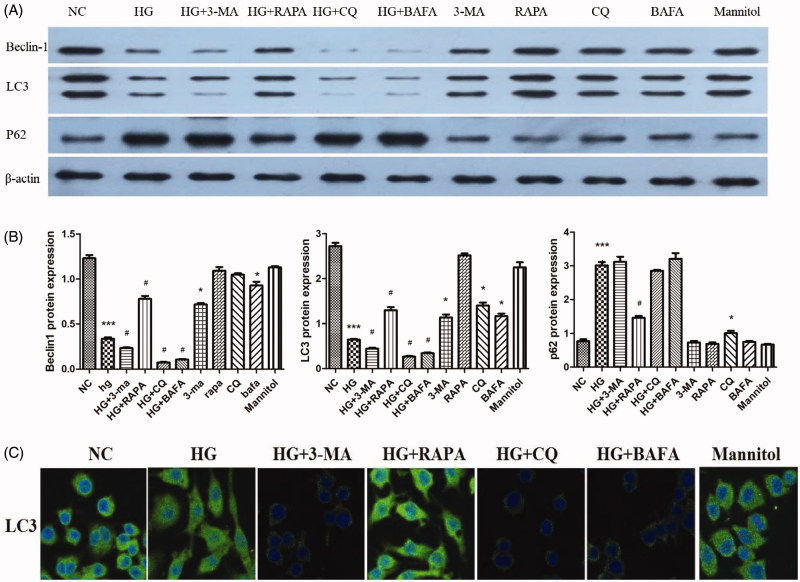
The autophagy levels of macrophages and the expression of autophagy markers. (A and B) The expression of Beclin-1, LC3, and P62 protein (Western blotting), *p<.05 versus NC, #*p*<.05 versus HG group, (*n* = 3). (C) The expression of LC3 protein shown by immunofluorescence. (×400; bars = 20 μm).

**Figure 3.**
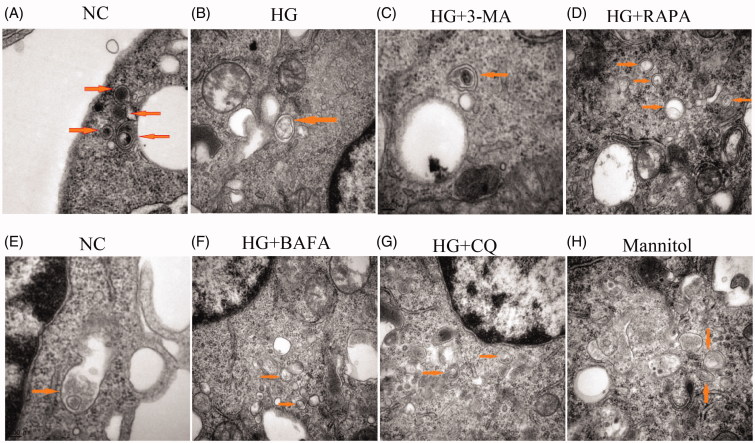
The change in the number of autophagosomes and degradation and fusion of autophagosome–lysosome by electron microscopy. (A–D) The arrows point to the autophagosome, representing the numbers of autophagosomes. (E–H) The arrows point to the autophagosome–lysosome, representing the process of degradation and fusion of autophagic lysosomes (×30 000).

**Figure 4.**
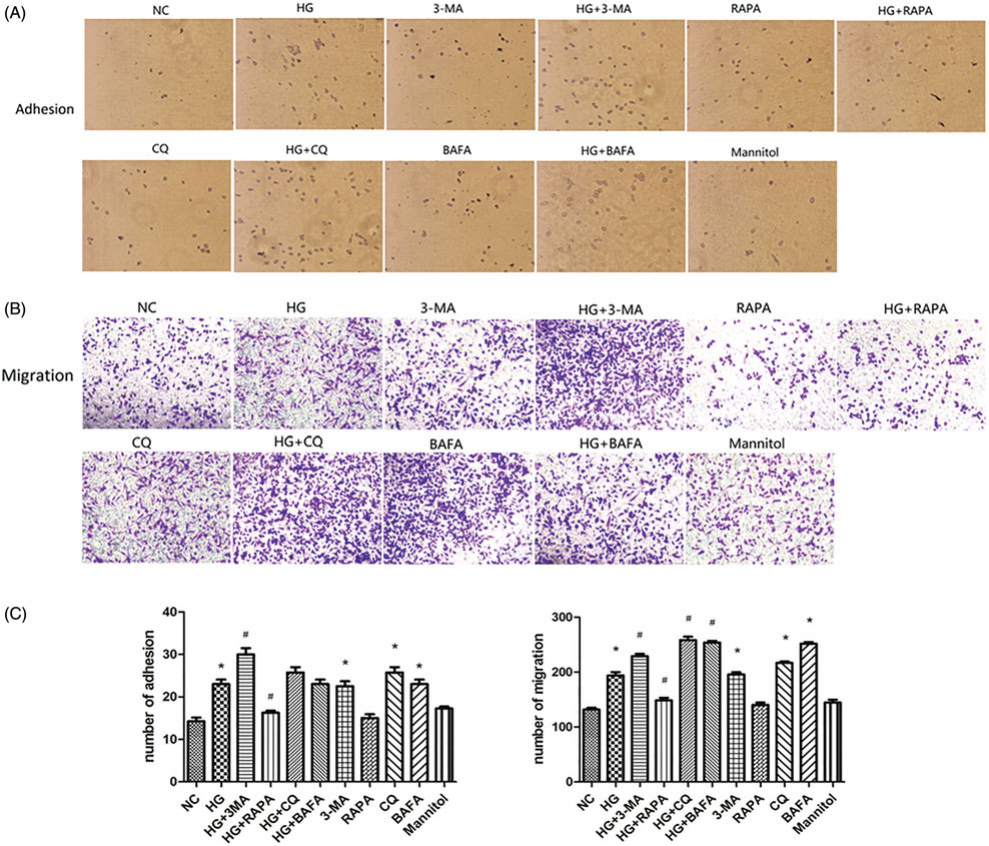
The adhesion and migration of macrophages. (A) The adhesion of macrophages under different intervention conditions (×200), *p<.05 versus NC, #*p*<.05 versus HG group, (*n* = 3). The migration of macrophages under different intervention conditions (×200), *p<.05 versus NC, #*p*<.05 versus HG group, (*n* = 3).

Western blotting showed that the expression of P62 in the HG group was increased compared with that in the NC group, and the expression of autophagy activity-related proteins LC3 and Beclin-1 was decreased (*p* < .05; Figure 2(A,B)). Immunofluorescence also showed that the expression of LC3 protein was lower in the HG group than that of the NC group (Figure 2(C)). Electron microscopy showed that the number of autophagosomes in the HG group was decreased compared with the NC group (Figure 3(A,B)). The numbers of adhesive and migratory macrophages in HG group were greater than those in NC group (*p* < .05; Figure 4(A,B)). Therefore, HG down-regulated the autophagy level and increased the number of adhesive and migratory macrophages.

### 3-MA, CQ, and BAFA inhibited autophagy levels and promoted adhesion and migration of macrophages

Compared with the NC group, Western blotting showed that the expression of P62 was increased in the CQ group, the expression of Beclin-1 was decreased in the 3-MA group, and the expression of LC3 was decreased in the 3-MA, CQ, and BAFA groups (*p* < .05; Figure 2(A,B)). In addition, the expression of P62 was further increased and the expression of LC3 and Beclin-1 were further decreased in HG + 3-MA, HG + CQ, and HG + BAFA groups compared with expression in the HG group (*p* < .05; Figure 2(A,B)). Immunofluorescence showed that the expression of the LC3 protein in HG + 3-MA, HG + CQ, and HG + BAFA groups was lower than that in the HG group (Figure 2(C)). Electron microscopy showed that the numbers of autophagosomes were decreased in the HG + 3-MA group compared with those in the NC group (Figure 3(A,C)). In addition, autophagosome-lysosome fusion was inhibited by BAFA compared with those in the NC group (Figure 3(E,F)), and autophagosome–lysosome degradation was inhibited by CQ (Figure 3(E,G)). The numbers of adhesive and migratory macrophages in the 3-MA, CQ, and BAFA groups were greater than those in the NC group (*p* < .05; Figure 4(A,B)). Compared with those in the HG group, the numbers of adhesive and migratory macrophages were further increased in the HG + 3-MA, HG + CQ, and HG + BAFA groups (*p* < .05; Figure 4(A,B)). Thus, 3-MA, CQ, and BAFA further aggravated the inhibition of the autophagy level of macrophages and increased the numbers of adhesive and migratory macrophages.

### RAPA activates autophagy, which reversed the numbers of macrophages adhesion and migration induced by HG

Western blotting showed that the expression of P62 was decreased in HG + RAPA group and the expression of LC3 and Beclin-1 was increased (*p* < .05; Figure 2(A,B)). Immunofluorescence results showed the expression of LC3 protein was increased compared with that of the high-glucose group (*p* < .05; Figure 2(C)). Compared with the HG group, electron microscopy results showed that the numbers of autophagosomes were increased in HG + RAPA group (Figure 3(B,D)). Compared with those in the HG group, the numbers of adhesive and migratory macrophages were significantly decreased induced by rapamycin treatment in the HG + RAPA group (*p* < .05; Figure 4(A,B)). Therefore, RAPA could up-regulate the macrophages autophagy level induced by HG and reduce the numbers of adhesive and migratory macrophages.

## Discussion

DN is a chronic inflammatory disease, characterized by inflammatory cell infiltration and pro-inflammatory factor overexpression [8,9]. Macrophage infiltration is an important histopathological feature in various chronic renal diseases including DN [10]. A large number of clinical and animal experimental studies have found that almost all of the patients with DN or animal kidney tissues are associated with different levels of macrophage infiltration [11,12]. The degree of macrophage infiltration is closely related to the severity of kidney damage [12–14]. Our previous studies have shown macrophage infiltration in the renal tissues of the STZ-induced DN rat [15]. Thus, preventing the macrophage infiltration is a key point for the treatment of DN. Macrophage infiltration is mediated by a variety of cell adhesion molecules and chemokine agents, which undergo rolling, adhesion, migration and other processes to reach tissues. The macrophages that reach the inflammatory site promote the development of renal inflammation and fibrosis [12,16,17]. Our research showed that when we stimulate macrophage with high glucose, the number of macrophages adhesion and migration was increased significantly. It implies a close relationship between macrophage adhesion migration and macrophage infiltration. Therefore, it is important to decrease numbers of macrophage adhesion and migration to alleviate macrophage infiltration in renal tissues.

Inflammation plays a pivotal role in pathophysiological processes of kidney diseases via releasing pro-inflammatory cytokines and infiltration of inflammatory cells [3]. Autophagy controls inflammation through regulatory interactions with innate immune signaling pathways, by removing endogenous inflammasome agonists and through effects on the secretion of immune mediators [18,19]. Autophagy also relies on the lysosomal degradation pathway. Since the autophagosome and lysosomes are fused to form autophagolysosomes, the encapsulated substances are degraded by lysosomal enzymes, which are called autophagolysosomal degradation pathways [20,21]. Macrophages adhesion and migration are closely related to autophagy level. Recent research demonstrates that selective autophagy mediated by the autophagy cargo receptor, NBR1, specifically promotes the dynamic turnover of integrin-based focal adhesion sites during motility. In this process, microtubules decompose partial adhesion focus, extracellular vesicle transport regulate change the stability of adhesion focus, and affecting the signal pathways related to focal adhesion, such as kinases FAK-src and calpain, which promote the decomposition and transfer of adhesion focus, ultimately determining the migration direction of cells [3,22]. Deretic V [23] found that autophagy contributes to antigen presentation and T cell homeostasis, and it affects T cell repertoires and polarization. This study not only showed the macrophage infiltration in the renal tissues of the STZ-induced DN rat, but also showed that the expression of LC3 and P62 (autophagy markers) in renal tissue decreased, suggesting that the level of autophagy in renal tissue of DN rats decreased. In cell experiments, the number of adhesion and migration of macrophages increased significantly and the autophagy level of macrophages was also inhibited when we stimulated macrophages with high glucose. This means that altering autophagy level of macrophage will affect the function of macrophage adhesion and migration. Therefore, regulating autophagy to affect macrophage adhesion and migration, maybe a key point to alleviate macrophage infiltration and prevent DN.

Xin [24] found that high glucose reduced the expression of LC3B-II and Beclin-1 in podocytes, suggesting that high glucose played a significant inhibitory role in autophagy. In our data, we find that high glucose can inhibit the formation of macrophage autophagosomes, which results in a decrease in autophagy levels and increases the amount of macrophage adhesion and migration.

Rapamycin (RAPA) is known as a kind of autophagy-induced medicine [25–27]. It can specifically bind to the target protein of rapamycin (mTOR) and inhibit the protein kinase activity of mTOR to consequently activate autophagy [28]. Recent research found that through AKT phosphorylation or activation, mTORC has also been reported to regulate cell migration [29,30]. In our data, we find that rapamycin can obviously increase the numbers of autophagosomes, recover the autophagy level inhibited by high glucose and reduce the amount of macrophage adhesion and migration. To further observe autophagosome formation in macrophage. We find found that 3-MA inhibits autophagosome formation in macrophages with or without pretreatment with high glucose, and the results are similar to those in the HG group. The previous theory suggested that 3-MA inhibits the activity of PI3K to down-regulate autophagy levels [27,31]. We consider that it may be related to the inhibition of cellular autophagosome formation. Therefore, we think that regulating the autophagosome formation can affect macrophage adhesion and migration.

Lysosome, as the final stage of the autophagy pathway, plays a vital role in the degradation of autophagy [32,33]. Bafilomycin A1 (BAFA), as a kind of vesicle type H + ATP inhibitor, not only inhibits the lysosomal activity but also blocks the fusion of the autophagosome and lysosome. Caixia [34] found that BAFA can increase the expression of P62 in podocytes. In our data, we found that BAFA inhibits the macrophage autophagy levels and observed that BAFA inhibits the fusion process of the autophagosome-lysosome in macrophages by electron microscopy. Chloroquine inhibits the process of degradation of the autophagosome-lysosome, blocking the normal process of autophagy. This inhibition leads to the accumulation of LC3 within the cell and inhibits the degradation of P62, which down-regulates the autophagy level [35,36]. Under the electron microscope, we observed that chloroquine inhibits the process of degradation of macrophage autophagosome-lysosome and decreases the expression of LC3 and Beclin-1 and increaseds the expression of P62. The above research shows that the fusion and degradation of autophagosome-lysosome play a vital role in maintaining autophagy activity and controlling macrophage adhesion and migration.

In conclusion, activation of autophagosome formation can increase autophagy levels and alleviate the adhesion and migration of macrophages. Decreasing autophagosome formation or inhibiting fusion and the degradation process of the autophagosome–lysosome can decrease autophagy levels and promote the adhesion and migration of macrophages. However, the mechanism of formation of the autophagosome and the fusion and degradation process of the autophagosome–lysosome need to be further explored.
